# Bilateral Persistent Pupillary Membrane

**DOI:** 10.1002/ccr3.71522

**Published:** 2025-11-20

**Authors:** Hassan Asadigandomani, Mohammad Soleimani

**Affiliations:** ^1^ Eye Research Center, Farabi Eye Hospital, Tehran University of Medical Sciences Tehran Iran; ^2^ Department of Ophthalmology University of North Carolina at Chapel Hill Chapel Hill North Carolina USA

**Keywords:** amblyopia, iris, persistent pupillary membrane, visual axis

## Abstract

A 31‐year‐old man presented for a routine eye exam with bilateral asymptomatic persistent pupillary membranes, with the central 1.5 mm zone of the pupil spared, preventing vision impairment.

## Key Clinical Message

Persistent pupillary membranes (PPMs) are common congenital remnants that typically do not interfere with visual development and rarely lead to amblyopia. Consequently, surgical intervention is seldom required. In this case, despite extensive bilateral PPMs, the spared visual axis and the patient's age beyond the amblyogenic period justified an observation‐only approach.

## 1 Case Presentation

A persistent pupillary membrane (PPM) is a congenital ocular anomaly, representing remnants of the anterior tunica vasculosa lentis, which nourishes the developing lens during embryogenesis [1, 2]. Most PPMs regress within the first year of life and require no intervention [2].

**FIGURE 1:**
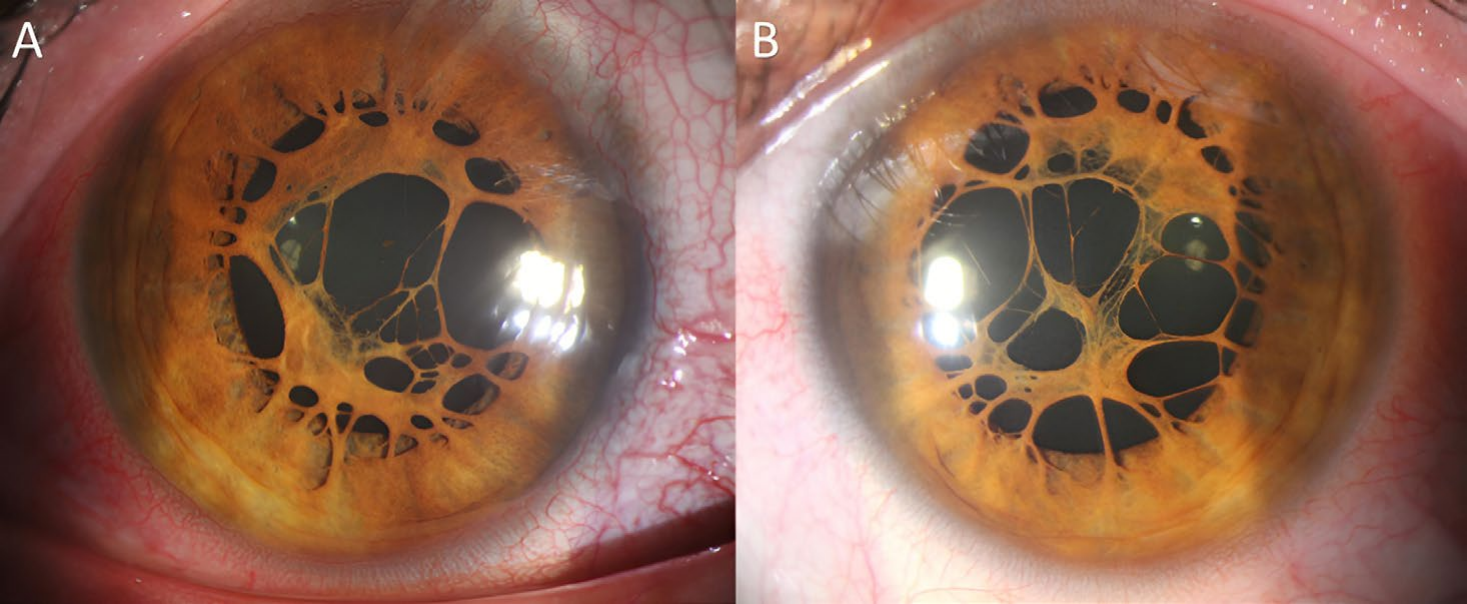
Slit‐lamp examination revealed extensive persistent pupillary membranes in the right (A) and the left eye (B). Fortunately, the 1.5 mm central zone of the pupil was not involved, preventing the development of amblyopia.

A 31‐year‐old healthy man presented for a routine eye examination. Uncorrected visual acuity (UCVA) was 20/20 in both eyes using a Snellen chart at 6 m, and best corrected visual acuity (BCVA) remained 20/20 bilaterally. Near visual acuity was Jaeger 1 (J1) in both eyes. Refraction was plano −0.75° × 120° for the right eye and plano −0.5° × 80° for the left eye. Biomicroscopy demonstrated transparent corneas, normal anterior chambers, and the absence of synechiae bilaterally. The gonioscopy findings and intraocular pressures (13 mmHg right eye, 14 mmHg left eye) were within normal limits. Slit‐lamp examination revealed extensive, radially oriented, and asymptomatic PPMs in both eyes (Figure 1A,B). The membranes measured approximately 2–3 mm in length, extending from the iris collarette toward the center but sparing the central 1.5 mm visual axis in both eyes. The dilated fundus examination was normal and unremarkable.

Importantly, the central 1.5 mm zone of the pupil, corresponding to the visual axis, was completely spared, resulting in preserved visual acuity and no signs of amblyopia. The sparing of the central 1.5 mm visual axis in both eyes prevented form‐deprivation amblyopia during the critical period of visual development. This underscores the importance of carefully evaluating the extent and location of PPMs, as peripheral involvement without axis obstruction may require no intervention [3].

## Author Contributions

**Hassan Asadigandomani:** data curation, writing – original draft, writing – review and editing. **Mohammad Soleimani:** conceptualization, data curation, writing – review and editing.

## Funding

The authors have nothing to report.

## Consent

Written informed consent was obtained, ensuring patient confidentiality and de‐identification.

## Conflicts of Interest

The authors declare no conflicts of interest.

## Data Availability

All data supporting the findings of this case image are included within the article.
